# A descriptive study of mastitis in Australian breastfeeding women: incidence and determinants

**DOI:** 10.1186/1471-2458-7-62

**Published:** 2007-04-25

**Authors:** Lisa H Amir, Della A Forster, Judith Lumley, Helen McLachlan

**Affiliations:** 1 Mother & Child Health Research, La Trobe University, Melbourne, Australia; 2 The Royal Women's Hospital, Melbourne, Australia; 3 Mercy Hospital for Women, Melbourne, Australia; 4 Clinical School of Midwifery and Neonatal Nursing Studies, La Trobe University, Australia

## Abstract

**Background:**

Mastitis is one of the most common problems experienced by women who are breastfeeding. Mastitis is an inflammation of breast tissue, which may or may not result from infection. The aims of this paper are to compare rates of mastitis in primiparous women receiving public hospital care (standard or birth centre) and care in a co-located private hospital, and to use multivariate analysis to explore other factors related to mastitis.

**Methods:**

Data from two studies (a randomised controlled trial [RCT] and a survey) have been combined. The RCT (Attachment to the Breast and Family Attitudes to Breastfeeding, ABFAB) which was designed to test whether breastfeeding education in mid-pregnancy could increase breastfeeding duration recruited public patients at the Royal Women's Hospital at 18–20 weeks gestation. A concurrent survey recruited women planning to give birth in the Family Birth Centre (at 36 weeks gestation) and women in the postnatal wards of Frances Perry House (private hospital). All women were followed up by telephone at 6 months postpartum. Mastitis was defined as at least 2 breast symptoms (pain, redness or lump) AND at least one of fever or flu-like symptoms.

**Results:**

The 6 month telephone interview was completed by 1193 women. Breastfeeding rates at 6 months were 77% in Family Birth Centre, 63% in Frances Perry House and 53% in ABFAB. Seventeen percent (n = 206) of women experienced mastitis. Family Birth Centre and Frances Perry House women were more likely to develop mastitis (23% and 24%) than women in ABFAB (15%); adjusted odds ratio (Adj OR) ~1.9. Most episodes occurred in the first 4 weeks postpartum: 53% (194/365). Nipple damage was also associated with mastitis (Adj OR 1.7, 95% CI, 1.14, 2.56). We found no association between breastfeeding duration and mastitis.

**Conclusion:**

The prevention and improved management of nipple damage could potentially reduce the risk of lactating women developing mastitis.

**Trial registration:**

Trial registration (ABFAB): Current Controlled Trials ISRCTN21556494

## Background

Mastitis is one of the most common problems experienced by women who are breastfeeding. Mastitis is an inflammation of breast tissue, which may or may not result from infection [1]. It is a painful, distressing condition which may require hospitalisation or lead to a breast abscess. Population-based studies in Australia, where breastfeeding initiation is over 80% and about 50% of women are breastfeeding at six months postpartum [2], have reported an incidence of mastitis in 15–20% of women in the six months postpartum [3-5]. Slightly fewer than 10% of American women experienced mastitis in three months postpartum in a large cohort study [6].

Factors associated with mastitis include milk stasis, nipple damage and maternal fatigue [1]. Some studies have suggested that women receiving private and birth centre care are more likely to experience mastitis than other women [7-9], however women giving birth in a birth centre and women with private insurance are more likely to initiate breastfeeding and to breastfeed for longer than women receiving public hospital care [10]. Does this explain the higher incidence of mastitis or are other factors involved? The aims of this paper are to compare rates of mastitis in primiparous women receiving public hospital care (standard or birth centre) and care in a co-located private hospital, and to use multivariate analysis to explore other factors related to mastitis.

## Methods

Data from two studies (a randomised controlled trial [RCT] and a survey) have been combined for this paper, reflecting a diverse range of women attending the Royal Women's Hospital, a public tertiary referral centre, and Frances Perry House (a private co-located hospital) in Melbourne, Australia. The Royal Women's Hospital is an accredited Baby Friendly hospital. Both the Royal Women's Hospital and Frances Perry House employ International Board Certified Lactation Consultants for inpatients and provide a breastfeeding clinic for women in the postnatal period.

Inclusion criteria in both studies included primiparity and ability to speak English. A RCT (Attachment to the Breast and Family Attitudes to Breastfeeding, ABFAB) to test whether breastfeeding education in mid-pregnancy could increase the duration of breastfeeding recruited public patients at the Royal Women's Hospital at 18–20 weeks gestation from May 1999 to August 2001 [11]. Participants in ABFAB were randomly allocated to a control group or one of two small-group interventions: a previously designed and tested tool to teach practical aspects of breastfeeding or an exploration of family attitudes to breastfeeding. Two groups of women not included in the randomised trial due to already high breastfeeding rates were recruited to a concurrent breastfeeding survey (Breastfeeding Survey of the Family Birth Centre and Frances Perry House or "the Survey"). The Survey recruited women planning to give birth in the Royal Women's Hospital's Family Birth Centre (recruited at 36 weeks gestation, August 2000 to March 2001) and women in the postnatal wards of Frances Perry House following the birth of their baby (November 2000 – March 2001). All women were followed up by telephone at six months postpartum. Data were collected using the same instruments in the two studies.

ABFAB aimed to recruit about 972 women based on identifying an increase in breastfeeding initiation from 75 to 85% and an increase from 38 to 52% in breastfeeding at six months [12]. Sample size for the Survey was based on an estimate that 20% of Australian women experience mastitis overall [4] and private/birth centre patients have an incidence of 25% [7,9] and public patients 15% (estimate): therefore a sample of 270 private/birth centre women for the Survey would be able to detect a difference between these women and public women in ABFAB with 95% confidence and 80% power. Sample size calculation was performed using EpiInfo 6. To allow for loss to follow up at six months, a sample size of 320 women for the Survey was planned.

Data collected at recruitment by self-administered questionnaire included a wide range of demographic factors as well as women's infant feeding intentions. At six months, we used a structured telephone interview to collect information about the duration of breastfeeding and breastfeeding problems. Women were asked "Can you estimate how old the baby was when you no longer experienced any nipple pain whilst feeding". Duration of nipple pain was stratified into pain lasted less than four weeks, pain lasted four weeks or more, and pain duration missing (if women gave a response that didn't fit either of these categories).

As there is no standard definition of mastitis, we asked women if they had any of the following symptoms: breast tenderness/pain, redness of any part of the breast, breast lump, a high temperature or flu-like symptoms, such as shivering, hot sweats or aches. If they answered 'yes' to any of the symptoms, they were asked further questions about timing and management of the episode. For the purposes of this study, mastitis was defined as "at least two out of the three breast symptoms (pain, redness, lump) AND at least one of fever or flu-like symptoms".

Nine hundred and eighty-one women were recruited into the three arms of the ABFAB trial (Practical skills, Attitudes and Standard care). As there were no differences in the background characteristics of the women in the three arms of the trial and no differences in outcome variables (initiation and duration of breastfeeding) [11,13], the results from the women in the three arms of the trial have been pooled and treated as a cohort.

Comparisons between the three groups of women (Family Birth Centre, Frances Perry House and the women in the ABFAB study) were conducted using chi-square tests, Student's t-tests, Kaplan-Meier survival estimates and log-rank tests. Incidence density of an illness (i.e. the number of cases occurring in people at risk of the illness) is the number of incident cases from time a to b divided by the number of person-units of experience observed from a to b [[14] p 91]. The incidence density of mastitis was calculated by dividing the number of episodes of mastitis in four week blocks by the number of completed weeks of women breastfeeding. Logistic regression was used to investigate the factors associated with mastitis. Independent variables to be examined were derived from the literature as well from clinical experience and discussions with colleagues. These included demographic variables (maternal age, education, marital status, private health insurance, family income, paid work/study), maternal characteristics (planned place of birth, smoking status), breastfeeding characteristics (cracked nipple, duration of nipple pain, maternal candida infection, oversupply of milk, duration of breastfeeding). Analyses were conducted using Stata 8.0.

Both studies received approval from the Human Research Ethics Committees at the Royal Women's Hospital and La Trobe University. The Survey was also approved by the Medical Advisory Committee at Frances Perry House.

## Results

One hundred and twenty-eight nulliparous women were recruited in the Family Birth Centre during antenatal visits out of a population of 142 (90% response rate). The population of primiparous women in Frances Perry House during the study period was 258 women of whom 238 were recruited. Thirty-four women who agreed to participate in the study and signed the consent form did not complete the recruitment questionnaire. Later, two women were found to be multiparous and were withdrawn from the study; therefore the total number of women eligible for follow-up was 202. Thus, 86% of those recruited completed the initial questionnaire (202/236), which represents 79% of the total eligible population (202/256).

The six month telephone interview was completed by 91% (889/981) of women in ABFAB, 87.5% (112/128) from the Family Birth Centre and 95% (192/202) from Frances Perry House. Thus, a total of 1193 women have data recorded for the six month interview.

Demographic details are presented in Table 1. The women in ABFAB were significantly younger (28.0 years) than women in the Family Birth Centre (29.6 years) and Frances Perry House (32.6 years)(p < 0.01). Women in ABFAB were less likely to have completed a degree (31%) than women in women in the Family Birth Centre (48%) and Frances Perry House (60%)(chi-square = 68.8, p < 0.01), and less likely to have a family income over A$50,000 per annum (42%) than women in the Family Birth Centre (50%) and Frances Perry House (87%)(chi-square = 134.5, p < 0.01). The method of birth was Caesarean section for 26% in ABFAB, 21% in Family Birth Centre and 42% in Frances Perry House (chi-square = 39.89, p < 0.01).

**Table 1:** Cha racteristics of women

**Characteristics**	**FBC (n = 112)**		**FPH (n = 192)**		**ABFAB (n = 889)**	
	n	%	n	%	n	%
**Age (mean, s.d.)**	29.6 (5.0)		32.6 (4.3)		28.5 (5.6)	
					Regression, p < 0.01	

**Marital status**						
Married	58	51.8	174	91.1	534	60.1
Living with partner	52	46.4	16	8.4	270	30.5
Not living with partner	1	0.9			45	5.1
Separated/divorced					2	0.2
Single	1	0.9	1	0.5	37	4.2
					Chi-sq = 90.9, p < 0.01	

**Highest education level**						
Completed primary school	16	14.3	15	7.9	211	23.8
Completed secondary school	42	37.5	62	32.5	403	45.5
Completed degree	54	48.2	114	59.7	272	30.7
					Chi-sq = 68.8, p < 0.01	

**Smoker**						
Yes	19	17.0	12	6.3	324	36.5
No	93	83.0	180	12	565	63.6
					Chi-sq = 78.6, p < 0.01	

**Family income**						
<$AUS20,000	9	8.0	1	0.5	130	14.7
$AUS20,001–50,000	42	37.5	16	8.4	325	36.7
>$AUS50,001	56	50.0	166	86.9	374	42.2
missing	5	4.5	8	4.2	57	6.4
					Chi-sq = 134.5, p < 0.01	

**Paid work/study (6 mo)**						
Work/study (full- or part-time)	67	35.1	49	43.8	273	30.9
Not at work/study	124	64.9	63	56.3	612	69.2
					Chi-sq = 8.01, p = 0.02	

**Method of birth**						
Vaginal	72	64.4	60	31.3	425	47.8
Forceps/vacuum	17	15.2	52	27.1	237	26.7
Caesarean section	23	20.5	80	41.7	227	25.5
					Chi-sq = 39.89, p < 0.01	

**Figure 1:**
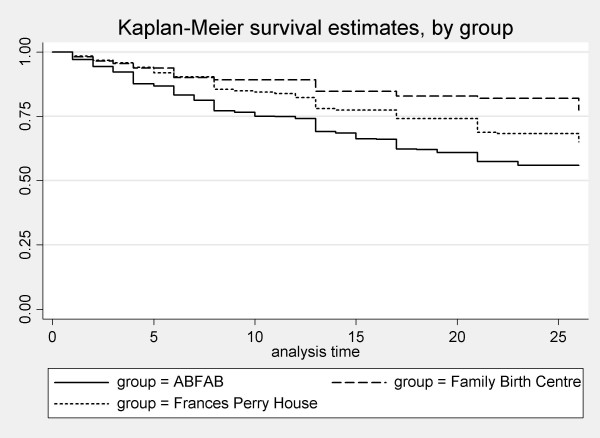
Duration of any breastfeeding, by group.

The breastfeeding outcomes are presented in Table 2. Seventy-seven percent of women in the Family Birth Centre group were breastfeeding at six months, which is significantly higher than women in the Frances Perry House group (63%) and women in the ABFAB study (53%) (chi-square = 26.9, p < 0.01). The mean duration of breastfeeding in the Family Birth Centre group was 22.7 weeks, 20.7 weeks in Frances Perry House group and 17.9 weeks in ABFAB (p < 0.01). The difference in breastfeeding duration (i.e. infant receiving any breast milk) between the women planning to give birth in the Family Birth Centre, Frances Perry House and ABFAB can be seen in the survival curves in Figure 1 (log-rank test, chi-square = 30.38, p < 0.01).

**Table 2:** Breastfeeding outcomes

**Characteristics**	**FBC (n = 112)**		**FPH (n = 192)**		**ABFAB (n = 889)**	
Mean duration of breastfeeding* (weeks, sd)	22.7 (7.5)		20.7 (8.5)		17.9 (9.8)	
					Regression, p = <0.01	

**Breastfeeding at 6 months**						
Breastfeeding at 6 mo	86	76.8	121	63.0	470	52.9
Not breastfeeding at 6 mo	26	23.2	71	37.0	419	47.1
					Chi-sq = 26.9, p < 0.01	

**Mastitis**						
Had mastitis	26	23.2	45	23.6	135	15.1
Didn't have mastitis	86	76.8	146	76.4	754	84.9
					Chi-sq = 10.6, p = <0.01	

**Cracked nipple**						
Had cracked nipple	16	14.3	28	14.6	106	11.9
No cracked nipple	96	85.7	164	85.4	783	88.1
					Chi-sq = 1.35, p = 0.5	

Duration of nipple pain** (weeks, sd) (missing = 118)	3.1 (3.8)		3.5 (3.8)		3.8 (5.4)	
					Regression, p = 0.5	

mo = months

*Data collected at 6 months, therefore actual duration would be longer.

**Includes women who had no nipple pain (n = 370).

### Mastitis results

Overall, 206/1193 (17.3%, 95%CI, 15.2%, 19.5%) women experienced mastitis as defined in this study. Women recruited in the Family Birth Centre and Frances Perry House were more likely to develop mastitis (23% and 24%) than women in ABFAB (15%); compared to ABFAB, women in Family Birth Centre had an odds ratio of 1.70 (95%CI, 1.06, 2.73) and Frances Perry House of 1.73 (95%CI 1.18, 2.53) of experiencing mastitis. There was no difference in the incidence of mastitis between the intervention and control arms of the ABFAB trial.

There were 365 episodes of mastitis in total; 194 episodes (53%) occurred in the first four weeks postpartum. Sixty-five episodes occurred in weeks five to eight and 44 episodes in weeks nine to twelve; thus 71% of episodes occurred in the first two months and 83% in the first three months postpartum.

**Figure 2:**
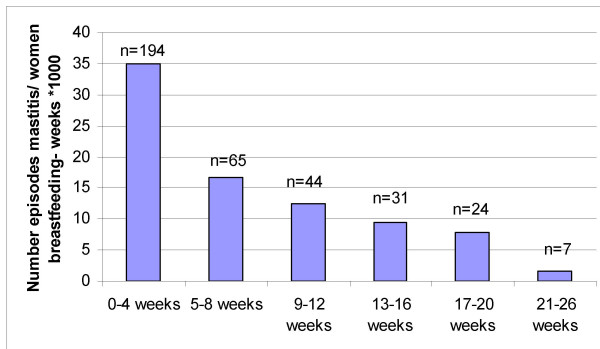
**Incidence of mastitis (n) by number of women breastfeeding-weeks**. n = number of cases of mastitis

The incidence density of mastitis can be seen in Table 3 and Figure 2. The highest density occurred in the first four weeks (35.0 episodes/number of women breastfeeding-weeks × 1000), was almost halved in the second four week period (16.6 episodes/number of women breastfeeding-weeks × 1000), and was down to 1.7 episodes/number of women breastfeeding-weeks × 1000 between 21 and 26 weeks postpartum.

**Table 3:** Incidence of mastitis by number of women breastfeeding-weeks

	**Number of episodes of mastitis**	**Number of women breastfeeding-weeks**	Number episodes/number women bf-weeks *1000
0–4 weeks	194	5550	35.0
5–8 weeks	65	3917	16.6
9–12 weeks	44	3556	12.4
13–16 weeks	31	3281	9.4
17–20 weeks	24	3021	7.9
21–26 weeks	7	4183	1.7

Total	365	23508	

**Figure 3:**
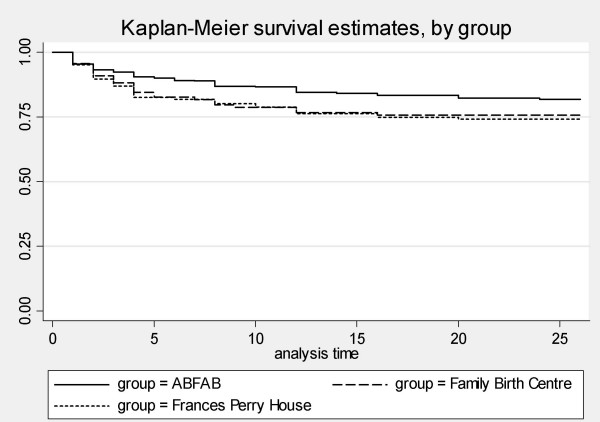
Timing of mastitis, by group.

A Kaplan-Meier survival curve has also been used to depict the timing of when women developed their first episode of mastitis in the three groups (Figure 3). Data from women who stopped breastfeeding before 26 weeks have been censored. The log-rank test shows a significant difference between the groups (chi2(2) = 7.76, p = 0.02).

**Figure 4:**
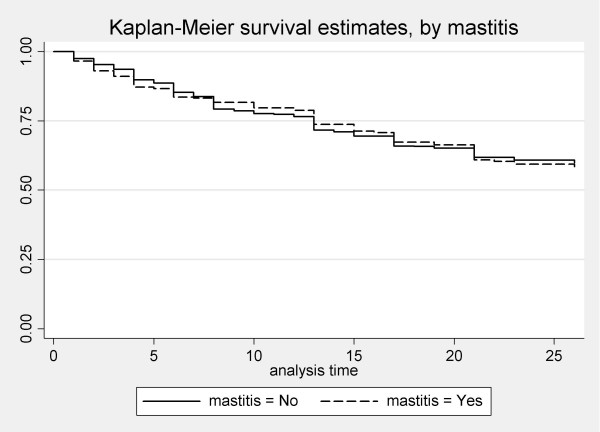
Duration of breastfeeding, by mastitis.

Survival analysis was performed to investigate whether there was a difference in duration of breastfeeding between women who developed mastitis and women who did not (Figure 4). There was no difference in duration of breastfeeding (log-rank test, chi2(1) = 0.08, p = 0.77).

Women who had stopped breastfeeding were asked the reason for stopping, however mastitis was rarely given as the reason women stopped breastfeeding. Eleven women in AFBAB cited "mastitis" and four gave "recurrent mastitis" as the main reason they stopped breastfeeding. Only one woman in the Survey cited "recurrent mastitis" as her main reason for stopping. Therefore sixteen women had stopped breastfeeding because of mastitis-related complaints (16/516), 3.1% of women who stopped breastfeeding before six months.

Of the 206 women with mastitis, 120 were continuing to breastfeed at six months postpartum (58%). Table 4 shows the timing of mastitis in relation to when women stopped breastfeeding. Thirty-six women stopped breastfeeding within three weeks of their first episode of mastitis. Women in ABFAB with mastitis were significantly more likely to stop breastfeeding within the following three weeks (29/134, 22%) than women in the Survey (7/71, 10%) (chi-square = 4.45, p = 0.04).

**Table 4:** Duration of breastfeeding following first episode of mastitis

	**ABFAB**	**FBC**	**FPH**	**Total**
**Women who stopped breastfeeding before 6 months**	n = 65	n = 7	n = 14	n = 86
Stopped bf before mastitis	5	1	2	8
Stopped within 3 wks	29	2	5	36*
Stopped between 3 and 26 wks	31	4	7	42

**Women breastfeeding at 6 months**	n = 70	n = 19	n = 31	n = 120
Mastitis in previous 3 wks	2	0	0	2
Mastitis > 3 wks earlier	68	19	31	118

**Total women with mastitis**				206

*chi-square = 4.45, p = 0.04

#### Complications of mastitis

Five women developed a breast abscess requiring aspiration and/or incision and drainage [15]. This represents 2.9% of women who took antibiotics for mastitis (95%CI 1.0, 6.7) or 0.4% of women who commenced breastfeeding (95%CI 0.14, 0.98) [15].

#### Other breastfeeding problems

The proportion of women with a cracked nipple (12–15%) was similar in the three groups (see Table 2). Only 230 women experienced no nipple pain while breastfeeding in the first six months. Including the women with no pain, the mean duration of nipple pain was 3.7 weeks (standard deviation 5.1) overall, with a median of 2 weeks.

### Multivariate analysis

A logistic regression model was developed to look at what factors were predictive of mastitis (the dependent variable). Independent variables were derived from the literature as well as from clinical experience and discussions with colleagues.

The independent variables were tested individually against the dependent variable and sorted into two groups: those to include in the preliminary model, and those to keep aside initially and be retested at a later stage. These univariate level tests were undertaken using logistic regression. Variables were entered in the model if the p-value of the Wald statistic was = 0.2 [16]. These included group (ABFAB, FBC or FPH), education, income (stratified in three levels), cracked nipple and duration of pain. Income was not found to be significant on univariate analysis, but was included in the model because income varied significantly between the groups. Although nipple thrush was significantly associated with mastitis, it was decided that the association may be due to thrush occurring following the episode of mastitis, and that it would not be appropriate to enter nipple thrush in this predictive model. Four women had missing data on education, and these four records were deleted, leaving 1189 records for analysis.

Variables were eliminated one at a time using logistic regression, only those with a p-value of the Wald statistic of = 0.05 were retained [16]. The process was repeated until only significant variables remained. At this stage all variables eliminated in the original univariate analysis were added back into the model one at a time to check that none had become significant given the reduced model. The only continuous variable, "duration of pain" was then checked for the correct parametric form using fractional polynomials [16]. The duration of pain was found to be non-linear and was dichotomised into pain in two levels (<four weeks and pain lasting four weeks or more). The only clinically/biologically plausible interactions of covariates was between cracked nipple and duration of pain. This was not retained in the model as the Wald statistic p-value = 0.22.

A "goodness of fit" test demonstrated a non-significant difference thus a good data fit (Hosmer-Lemeshow chi2(4) = 0.66, p = 0.96). The sensitivity of the model, that is, how often it correctly predicted the outcome (y) given the value of a covariate (x) was tested using the area under the ROC curve. To be said to have good discrimination, a model should have a statistical value of ≥ 0.7 from the ROC test, that is the area under the ROC curve should be large so that for every increase in specificity you move closer to a high sensitivity [16]. The lroc test identified that the area under the ROC curve was 0.6195. The lstat test showed 82.67% were correctly classified.

The final model is presented in Table 5. Family Birth Centre and Frances Perry House women had an increased odds of experiencing mastitis (AdjOR~1.9) compared to women receiving public hospital care. The presence of a cracked nipple was also associated with mastitis (AdjOR 1.7, 95%CI, 1.14, 2.56).

**Table 5:** Mastitis: Multivariate analysis

	**Univariate analysis**			**Multivariate analysis**			
	**Odds Ratio**	**(95% CI)**		**Adjusted Odds Ratio**	**(95% CI)**		**P**

**Group**							
ABFAB (Ref)	1			1			
FBC	1.70	1.05	2.70	1.89	1.16	3.08	0.011
FPH	1.73	1.18	2.53	1.86	1.25	2.76	0.002

**Cracked nipple**							
No (Ref)	1			1			
Yes	1.92	1.29	2.86	1.71	1.14	2.56	0.010

**Pain**							
<4 weeks (Ref)	1			1			
≥ 4 weeks	1.73	1.27	2.36	1.58	1.15	2.19	0.005
Pain duration missing				0.61	0.33	1.13	0.113

ABFAB = Attachment to the Breast and Family Attitudes to Breastfeeding trial; FBC = Family Birth Centre group; FPH = Frances Perry House (private hospital) group. Ref = Reference

## Discussion

Fifty-seven percent (677/1193) of women in this study overall were breastfeeding at six months postpartum which is higher than the proportion of 45% reported for the State of Victoria at that time [17]. Women in the Family Birth Centre and Frances Perry House groups had higher proportions of women breastfeeding at six months, 77% and 63% respectively, than women in ABFAB, 53%. This is expected, as women attending the Family Birth Centre and Frances Perry House were likely to be older and more educated than women in ABFAB, factors that are known to be associated with longer duration of breastfeeding [10].

Mastitis is a continuum from a mild inflammatory condition to a severe bacterial infection [18]. We used a strict definition of mastitis in these studies in order to estimate the proportion of breastfeeding women who experienced a clinically significant illness. We avoided asking about mastitis directly, by collecting information about mastitis symptoms, in order to reduce bias. Using our definition, 17% of women experienced at least one episode in the six months after birth, which is similar to estimates of about 20% in other Australian studies [4,5,19]. As others had found, more women in the birth centre group (23%) and private patients (24%) had mastitis than women receiving standard public hospital care (ABFAB, 15%) [7-9].

Approximately three-quarters of the episodes of mastitis occurred in the first eight weeks postpartum. Women with mastitis were not more or less likely to continue breastfeeding than other women. In contrast, Vogel and colleagues found in a New Zealand study that women with mastitis were likely to breastfeed for longer than women without mastitis [20].

We were interested in examining the variables associated with mastitis using logistic regression. Although our hypothesis was that longer duration of breastfeeding may be associated with mastitis, we found no association between breastfeeding duration and mastitis, therefore duration was not entered into the model. The presence of a cracked nipple and longer duration of nipple pain were associated with mastitis on both univariate and multivariate analysis. Although maternal education was significantly associated with mastitis on univariate analysis, this was no longer associated with mastitis on multivariate analysis. Women in both the Family Birth Centre and Frances Perry House (private patients) had an increased odds of experiencing mastitis (OR~1.9) compared to women receiving public hospital care. This finding is consistent with earlier studies [7-9], but the reason for this association is still unclear. It is possible that women with private insurance differ from public patients in their breastfeeding behaviour – it may be that they are more likely to regulate intervals between feeds in order to establish a "routine". Private patients tend to have longer hospital stays than public patients [21] which could increase the transmission of pathogens to new mothers and babies from hospital staff. We consider this is unlikely as women attending the Family Birth Centre generally have short stays (twenty-four hours) and they had a higher risk of mastitis than public patients receiving standard care.

There are a number of limitations to this study. Firstly, it would have been preferable to collect information about mastitis on several time points, however we had to rely on one interview at six months postpartum as this was the study design of the RCT. More information could have been collected about breastfeeding patterns: data on how often women fed and the length of intervals between feeds overnight could have been useful.

When looking for factors that could be varied in order to reduce or prevent mastitis in lactating women it is obvious that demographic factors cannot be changed. It is clear that damage to nipples is an important factor in the development of mastitis; presumably the damaged skin allows bacteria to enter the nipple resulting in mastitis. Many other authors have also reported this association between damaged nipples and mastitis [6,22-24]. The prevention of damaged nipples and improved management of damaged nipples could potentially reduce the risk of lactating women developing mastitis.

## Competing interests

The author(s) declare that they have no competing interests.

## Authors' contributions

LHA designed and conducted the Survey, analysed the data and drafted the paper. DAF and HM conducted the RCT and analysed the data. JL supervised the conduct and analysis of the Survey and the RCT. All authors approved the final draft.

## Pre-publication history

The pre-publication history for this paper can be accessed here:


